# The chain mediating role of family health and physical activity in the relationship between life satisfaction and health-promoting lifestyles among young adults in China

**DOI:** 10.3389/fpubh.2024.1408988

**Published:** 2024-09-04

**Authors:** ZhaoZhi Liu, Li Huang, HaoDong Tian, HaoWei Liu, HaoYue Luo, YunFei Tao, Li Peng

**Affiliations:** College of Physical Education, Southwest University, Chongqing, China

**Keywords:** life satisfaction, family health, physical activity, health-promoting lifestyle, young adult, China, chain mediation

## Abstract

**Background:**

Unhealthy lifestyles during adolescence are significant factors leading to chronic diseases in the future. Enhancing health-promoting lifestyles among young adults in China is crucial for preventing and reducing the risk factors of chronic diseases.

**Objective:**

This study aims to explore the relationships between life satisfaction, family health, physical activity, and health-promoting lifestyles among young adults in China. It also seeks to confirm the chain mediation role of family health and physical activity in the influence of life satisfaction on health-promoting lifestyles in this population.

**Methods:**

This study, conducted from August 2023 to November 2023, employed a random sampling method to recruit young adult participants aged 18–40 in the southwestern region of China. Variables were measured using the Satisfaction with Life Scale (SWLS), the Family Health Scale-Short Form (FHS-SF), the Physical Activity Rating Scale-3 (PARS-3), and the Health-Promoting Lifestyle Profile II Revised (HPLP-IIR). Data analysis was performed using SPSS 27.0 and the PROCESS macro version 4.1.

**Results:**

The results indicated that life satisfaction was positively correlated with family health (*r* = 0.225), physical activity (*r* = 0.245), and health-promoting lifestyles (*r* = 0.506). Family health was positively correlated with physical activity (*r* = 0.320) and health-promoting lifestyles (*r* = 0.312). Physical activity was positively correlated with health-promoting lifestyles (*r* = 0.429). Additionally, life satisfaction could influence health-promoting lifestyles directly (effect = 0.369) and through three mediation pathways: (a) family health (effect = 0.033); (b) physical activity (effect = 0.050); (c) family health and physical activity (effect = 0.020).

**Conclusion:**

This study supports the mediating role of family health and physical activity in the influence of life satisfaction on health behaviors among young adults in China. Therefore, we recommend that future public health initiatives place greater emphasis on family health and create conditions that facilitate physical activity for this group. This could be an important direction for further enhancing health-promoting lifestyles among young adults in China.

## 1 Introduction

Over the past few decades, China has undergone rapid development in social, economic, and educational sectors, significantly bolstering its comprehensive national strength. However, this accelerated growth has also introduced a series of challenges, including increased pressure on employment, intensified competition for resources, and a rise in the cost of living (1, 2). Young adults, as the backbone of societal progress, are particularly susceptible to these issues. Consequently, a significant portion of this demographic may sacrifice rest and even health to remain competitive, exemplified by the widespread adoption of the so-called “996” working schedule, which entails 6 days of work per week, from 9 a.m. to 9 p.m. daily (3). Under the impact of this mental strain and work pattern, there might be detrimental effects on their psychophysical health, potentially leading to negative repercussions on their social life and family relations, hence diminishing their life satisfaction (4). Health-promoting lifestyle constitutes an engaged and intentional mode of living, distinguished by individuals partaking in deliberate behaviors designed to preserve or augment their health (5, 6). Current research on health-promoting lifestyle has predominantly concentrated on children, adolescents, or older adults, to some extent neglecting young adults. Previous studies have indicated a bidirectional relationship between health-promoting lifestyles and individuals’ life satisfaction (7–9). This bidirectional pathway of influence may represent a significant direction for enhancing the psychophysical health of young adults in China. Life satisfaction, as a facet of subjective well-being, along with health-promoting lifestyles (HPL), has consistently been a focal point of research within the fields of public health and health sciences (10). Building upon the foundation of existing research findings, we hypothesize that an enhancement in life satisfaction may positively influence an individual’s engagement in health-promoting lifestyles. Additionally, to further comprehend the intrinsic mechanisms of this process, we acknowledge the potential mediating roles played by family health status and the level of physical activity (11, 12). Accordingly, this study aims to investigate how life satisfaction affects the health-promoting lifestyles of young adults and endeavors to unveil the mediating roles of family health and physical activity. This research is committed to offering practical references and a theoretical foundation for enhancing health-promoting lifestyle among the young adult population in China.

Life satisfaction was defined as a cognitive and global assessment of an individual’s overall quality of life (13). The rapid pace of societal development and multifaceted pressures in China often compel young adults to devote a considerable amount of energy and time to their careers (14), driven by high life expectations, they frequently sacrifice their leisure time and even their health (15). This sacrifice can lead to physical and mental health issues, which directly diminish their life satisfaction, potentially creating a vicious cycle. Research indicates that a high level of life satisfaction can result in more positive emotional experiences and can also lead to behavioral changes in individuals (16). Life satisfaction not only has subjective implications for an individual but is also closely linked to alterations in their behavior. Therefore, the relationship between life satisfaction and individual behavior is one of the key areas to address among young adults in China. Previous evidence suggests that the risks of cardiovascular diseases, depression, and sudden death are directly related to an individual’s lifestyle (17–19). Considering the impact of life satisfaction on individual behavior, it is meaningful to explore whether there is a promotive effect between life satisfaction and individual health behaviors. Although past research has primarily focused on the positive impact of health-promoting lifestyles on life satisfaction (20), there is growing evidence suggesting that the relationship between life satisfaction and health-promoting lifestyles may be bidirectional (7, 20, 21), This means that individuals with higher life satisfaction tend to engage in higher levels of health-promoting lifestyles, a concept that is also reflected in studies within the fields of psychology and public health (7, 22). Therefore, we propose Hypothesis 1 (H1): Life satisfaction can positively predict the health-promoting lifestyle practices of the young adult.

In exploring the mechanisms through which life satisfaction influences health-promoting lifestyles among young adults in China, the impact of the family environment on individual cognition and behavior is noteworthy. Within the context of traditional Chinese culture, family-based social relationships play an essential role in individual development (23). Particularly since China implemented the “family planning” and “late marriage and late childbirth” policies in 1979, contemporary young adults are mostly single children, which undoubtedly amplifies the influence of the family on the individual (24).Family health refers to the comprehensive health status of family members, encompassing physical, psychological, and social relational well-being, and reflects the functional state and quality of life of the entire household (25). Investigations into family health within the extant literature are limited; nevertheless, family health exerts a significant influence on individual behavior as well as psychophysical well-being (26, 27), this relationship has gradually come to the attention of scholars in recent years (28). A cross-sectional study indicates that there is a significant correlation between family health and the health perceptions and behaviors of family members (29). Further evidence suggests that individuals with higher levels of life satisfaction demonstrate greater psychological resilience (30), which affords them a more positive attitude when confronting life’s challenges. This positive disposition can be conveyed to other family members (31). In other words, higher life satisfaction may influence individual health behaviors through positive family health, suggesting that family health could play a significant mediating role in the process by which life satisfaction impacts the health-promoting lifestyles of young adults. So, we propose H2: Family health mediates the relationship between life satisfaction and the practice of health-promoting lifestyles among young adults.

Physical activity is defined as any bodily movement produced by the contraction of skeletal muscles that results in energy expenditure (32). Previous studies have shown that physical activity not only brings physiological benefits but also helps alleviate anxiety, relieve psychological stress, and promote interpersonal relationships (33). The close association between life satisfaction and participation in physical activities has been confirmed by research (34). According to the Health Behavior Model, high life satisfaction may increase an individual’s motivation for physical activity, which in turn enhances their physical and mental health, further promoting their level of health-promoting lifestyles (35). The Self-Determination Theory also suggests that satisfying psychological needs can stimulate self-regulated behaviors such as physical activity, which in turn improve overall health status (36).This view is increasingly supported by research (37–39). Therefore, we propose Hypothesis 3 (H3): Physical activity mediates the relationship between life satisfaction and health-promoting lifestyles among young adults.

**Figure 1:**
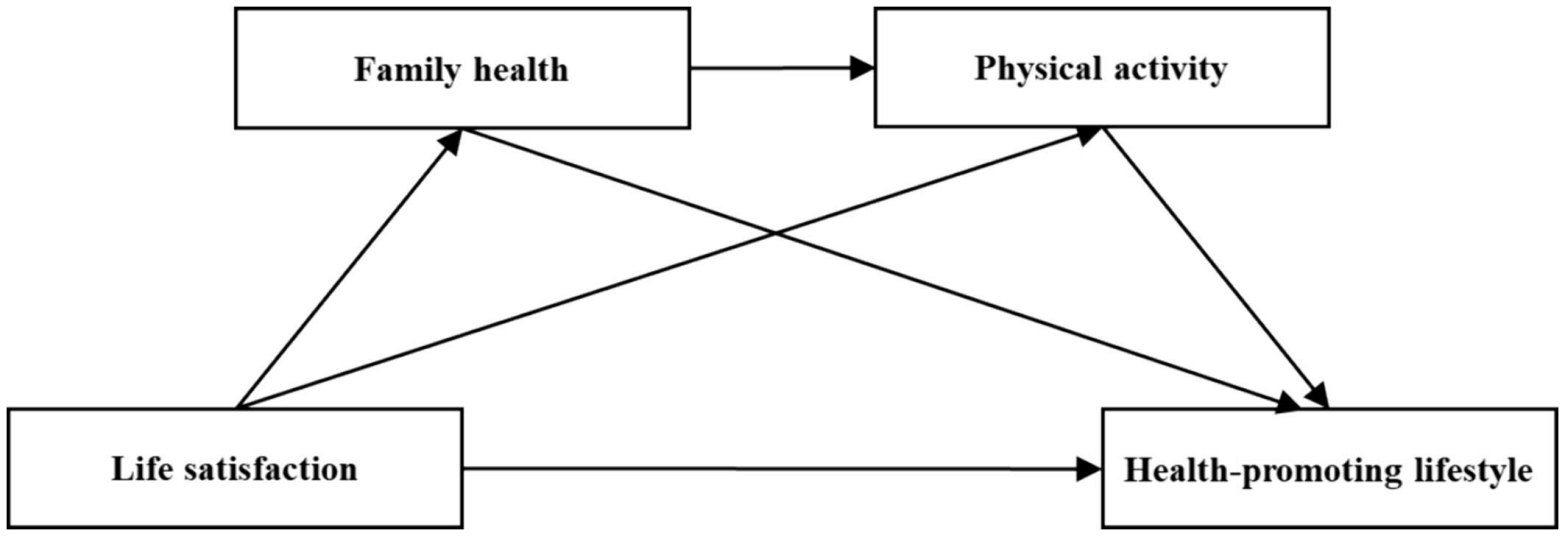
The model of the mediator role of family health and physical activity between life satisfaction and health-promoting lifestyle.

As previously mentioned, family health and physical activity may both play a mediating role in the process by which life satisfaction influences health-promoting lifestyles among young adults. We have also uncovered some evidence that suggests the potential existence of a serial mediation effect involving family health and physical activity in this process. Sallis and Nader (40)indicate that individuals with higher influence within a family can affect the health behaviors of other family members. Notably, their research identifies that physical activity may play a facilitative role in this process. Additionally, evidence from Brazil indicates that the family plays a crucial role in adolescent participation in physical activity (41), this suggests that family health and physical activity likely have a serial mediating effect in the process between life satisfaction and health-promoting lifestyle among young adults. Therefore, we propose H4: Family health and physical activity act as a serial mediator in the relationship between life satisfaction and health-promoting lifestyle among young adults. The theoretical model of this study is presented in Figure 1.

## 2 Methods

### 2.1 Study design and participates

This is a cross-sectional study. Between August 2023 and November 2023, we used a random sampling method to recruit participants in the southwestern regions of China (Chongqing, Sichuan, Yunnan, and Guizhou) through both online and offline methods. Since the participants were from the Chinese population, the questionnaire was administered in Chinese. The research instruments we used were scientifically translated and validated scales, suitable for the young adult population in China, with reliable validity and reliability. The exclusion criteria were: (a) questionnaires from respondents under 18 or over 40 years old; (b) questionnaires with a response time of less than 300 s; (c) questionnaires that were not fully completed; (d) questionnaires with patterned responses; (e) questionnaires with logical inconsistencies. A total of 774 valid questionnaires were included, with a response rate and valid questionnaire rate of 93.34 and 86.6%, respectively. This study was conducted in strict accordance with the 1964 Helsinki Declaration and its subsequent amendments. Additionally, our research was approved by the Ethics Committee of Southwest University Hospital (SWU-ETF-2023-07-17-011).

### 2.2 Measures

#### 2.2.1 Satisfaction with life scale

Satisfaction with Life Scale (SWLS) was used to assess an individual’s contentment with their general life circumstances. This scale has been revised by Xiong and Xu (42) and is a unidimensional tool that comprises five items. Each item is rated on a 7-point Likert scale, from “strongly disagree” (1 point) to “strongly agree” (7 points). The overall score can range from 5 to 35, with higher scores reflecting higher levels of life satisfaction. In this study, the Cronbach’s α coefficient for the scale was 0.854.

#### 2.2.2 Health-promoting lifestyle profile-II revised

Health-Promoting Lifestyle Profile-II Revised (HPLP-IIR) was used to measure the extent to which individuals engage in behaviors conducive to health promotion. This instrument, culturally adapted by Cao et al. (43), comprises six dimensions: Interpersonal Relations (5 items), Nutrition (6 items), Health Responsibility (11 items), Physical Activity (8 items), Stress Management (5 items), and Spiritual Growth (5 items), totaling 40 items. Within the scale, each item is rated using a 4-point Likert scale, with options including “Never” (1 point), “Sometimes” (2 points), “Often” (3 points), and “Always” (4 points). The total score ranges from 40 to 160. Scores from 40 to 80 are considered a low level of health-promoting lifestyle, 81 to 120 a moderate level, and 121 to 160 a high level. In this study, the Cronbach’s α coefficient for the scale was 0.956.

#### 2.2.3 Family health scale short form

Family Health Scale Short Form (FHS-SF), translated and adapted by Wang et al. (44), was used to assess an individual’s family health functioning. The scale includes four dimensions: Emotional Health (3 items), Family Health Behaviors (2 items), Family Health Resources (3 items), and Family Social Support (2 items). This scale uses a 5-point Likert scoring system, from “Strongly Disagree” (1 point) to “Strongly Agree” (5 points), with three items reverse-scored and the remainder positively scored. The total score ranges from 10 to 50 points, with higher scores indicating better family health status. In this study, the Cronbach’s α for the scale was 0.746.

#### 2.2.4 Physical activity rating scale

Physical Activity Rating Scale-3 (PARS-3) was used to assess an individual’s level of physical activity (45). PARS-3 consists of three items: exercise intensity, exercise duration, and exercise frequency. Each item is divided into five levels, corresponding to scores from 1 to 5. The total score for the scale is calculated using the formula: frequency score × (duration score - 1) × intensity score, with a possible range of 0 to 100. Higher scores indicate a higher level of physical activity. In this study, the Cronbach’s α for the scale was 0.705.

### 2.3 Statistical analysis

IBM SPSS 27.0 was used for statistical analysis. Descriptive statistics were first conducted, using frequencies and percentages to describe the demographic characteristics of the participants. Cronbach’s alpha values were used to represent internal consistency coefficients and reliability. Pearson correlation analysis was performed for the independent variables, dependent variable, and the two mediating variables. Harman’s single-factor test was used to check for common method bias among all variables. Chain mediation effect analysis was conducted using the SPSS PROCESS v4.1 macro program compiled by Hayes (46). In PROCESS, Model 6 was used for mediation analysis, with 5,000 bootstrap resamples, and the bias-corrected percentile bootstrap confidence interval (CI) was used to evaluate the effect size. Mediation effects were considered significant when the 95% CI did not include zero (47). Additionally, gender, employment status, and marital status were included as covariates in the model during the analysis.

## 3 Results

### 3.1 Common method deviation test

In this study, Harman’s single-factor test was applied to all variables during the exploratory factor analysis. This analysis unveiled that there was a total of nine factors with eigenvalues greater than one. Notably, the largest factor accounted for only 30.91% of the variance, which is less than the 40% threshold (48), suggesting that method bias is not a significant concern in the dataset under consideration.

### 3.2 Descriptive statistics and correlation analysis

A total of 894 questionnaires were collected for this study. After applying the exclusion and inclusion criteria, 774 valid questionnaires were included for data analysis. Most respondents were male (69.0%); the largest age group was 18–24 years (40.3%), followed by 25–29 years (29.2%); the majority of respondents were single (62.8%). Detailed demographic information of the participants is presented in Table 1.

**Table 1:** Characteristics of participates.

Categorical variables	Category	*N*	Percentage (%)
Gender	Male	534	69.0
	Female	240	31.0
Age group (years)	18 ~ 24	312	40.3
	25 ~ 29	226	29.2
	30 ~ 34	131	16.9
	35 ~ 39	105	13.6
Marital	Single	486	62.8
	Married	275	35.5
	Divorce	13	1.7
Local	Rural	619	80.0
	City	155	20.0
Employment	Unemployed	288	41.1
	Employed	456	58.9

The results of the Pearson correlation analysis (Table 2) show that life satisfaction is positively correlated with family health (*r* = 0.255, *p* < 0.01), physical activity (*r* = 0.245, *p* < 0.01), and health-promoting lifestyle (*r* = 0.506, *p* < 0.01). Family health is positively correlated with physical activity (*r* = 0.320, *p* < 0.01) and health-promoting lifestyle (*r* = 0.312, *p* < 0.01). Finally, physical activity is positively correlated with health-promoting lifestyle (*r* = 0.429, *p* < 0.01). These results provide preliminary support for further validation of the chain mediation effect.

**Table 2:** Descriptive statistics and correlation analysis.

Variables	*M*	SD	SWLS	FHS-SF	PARS-3	HPLP-IIR
SWLS	22.34	5.64	1			
FHS-SF	35.87	5.92	0.255**	1		
PARS-3	28.48	22.98	0.245**	0.320**	1	
HPLP-IIR	109.18	17.46	0.506**	0.312**	0.429**	1

M, Mean; SD, Standard deviation; **p < 0.01.

### 3.3 Testing the mediating effects of family health and physical activity

Table 3 presents the detailed results of regression analyses, indicating that life satisfaction positively predicts young adults’ health-promoting lifestyle (β = 0.472, *p* < 0.001), thus confirming H1. When mediators were included in the analysis, life satisfaction significantly predicted family health (β = 0.225, *p* < 0.001) and physical activity (β = 0.189, *p* < 0.001). Family health significantly predicted both physical activity (β = 0.286, *p* < 0.001) and health-promoting lifestyle (β = 0.129, *p* < 0.001). Additionally, physical activity was a significant positive predictor of health-promoting lifestyle (β = 0.267, *p* < 0.001). The direct effect of life satisfaction on health-promoting lifestyle was reduced after including the mediators (β = 0.369, *p* < 0.001). These findings suggest that there is a significant mediating and chain mediation effect of family health and physical activity on the impact of life satisfaction on health-promoting lifestyle.

**Table 3:** Regression analysis of Chain mediation effects for family health and physical activity.

Regress equation		Fitting index			Significance		
Outcome variable	Predictor variable	*R*	*R* ^2^	*F*	β	SE	*t*
Health-promoting lifestyle	Life satisfaction	0.524	0.275	72.764^***^	0.472	0.029	16.493^***^
	Gender				−0.009	0.059	−0.148
	Employment				−0.085	0.040	−2.099^*^
	Marital				−0.165	0.055	−2.987^**^
Family health	Life satisfaction	0.302	0.091	19.239^***^	0.225	0.035	7.266^***^
	Gender				−0.067	0.072	−0.924
	Employment				0.139	0.050	2.800^**^
	Marital				0.166	0.068	2.451^*^
Physical activity	Life satisfaction	0.436	0.190	36.094^***^	0.189	0.036	5.276^***^
	Family health				0.286	0.036	8.061^***^
	Gender				−0.505	0.071	−7.111^***^
	Employment				0.026	0.049	0.531
	Marital				−0.139	0.067	−2.072^*^
Health-promoting lifestyle	Life satisfaction	0.629	0.396	83.864^***^	0.369	0.028	12.418^***^
	Family health				0.129	0.028	4.628^***^
	Physical health				0.267	0.027	9.782^***^
	Gender				0.140	0.055	2.522^*^
	Employment				−0.120	0.037	−3.243^**^
	Marital				−0.162	0.051	−3.191^**^

*N* = 744, SE = standard error, β = effect value, *p < 0.05, **p < 0.01, ***p < 0.001.

**Figure 2:**
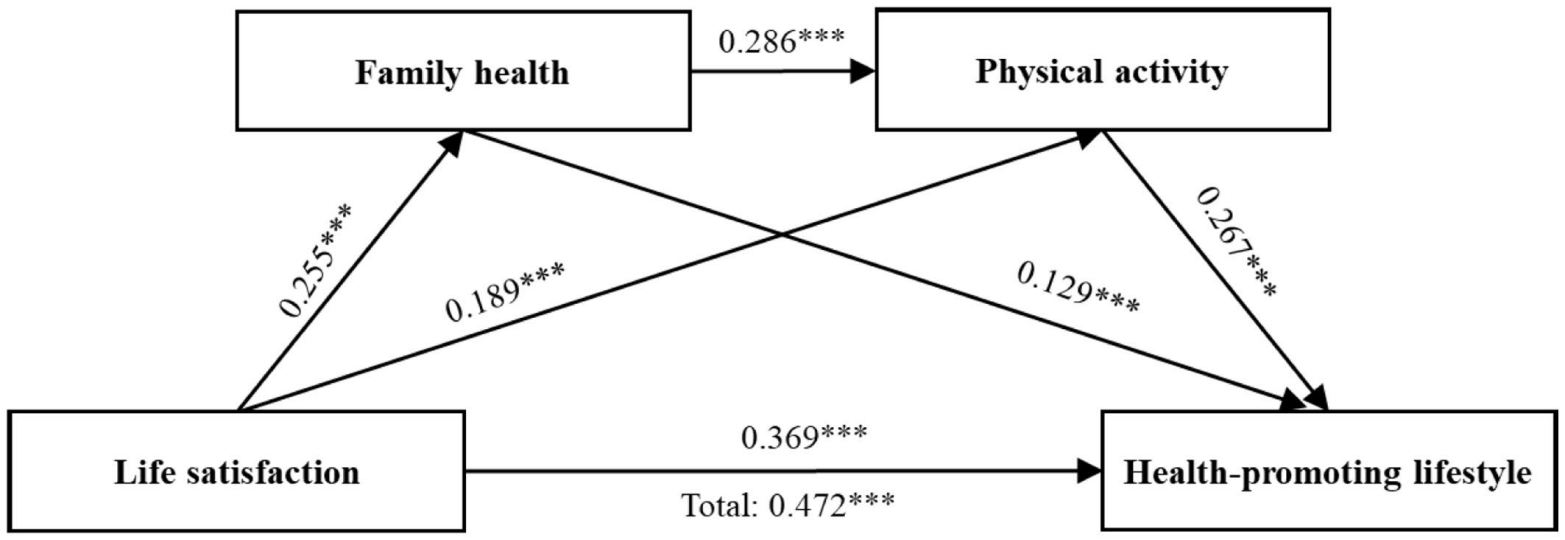
Mediating effect analysis of family health and physical exercise between life satisfaction and health-promoting lifestyle. ^***^p < 0.001.

Figure 2 and Table 4 demonstrate the mediating effect and the chain mediation model for each path within the mediation model. The mediation effect consists of three indirect paths: Path 1: Life satisfaction → Family health → Health-promoting lifestyle (Effect = 0.033, 95% CI [0.017, 0.052]), Path 2: Life satisfaction → Physical activity → Health-promoting lifestyle (Effect = 0.050, 95% CI [0.028, 0.075]), and Path 3: Life satisfaction → Family health → Physical activity → Health-promoting lifestyle (Effect = 0.020, 95% CI [0.012, 0.028]). The proportions of the indirect effects to the total effect for the three paths are 6.99, 10.59, and 4.23%, respectively. The 95% confidence intervals for these indirect effects do not include 0, indicating that all paths’ mediation effects are significant (47). These results support Hypotheses 2–4.

**Table 4:** The mediation effect of family health and physical activity (*n* = 774).

Model	Effect	SE	Bootstrap CI LL	Bootstrap CI UL	Efficiency ratio
Direct effect	0.369	0.028	0.315	0.423	78.18%
Total indirect effect	0.103	0.015	0.074	0.133	21.82%
Indirect effect 1	0.033	0.009	0.017	0.052	6.99%
Indirect effect 2	0.050	0.012	0.028	0.075	10.59%
Indirect effect 3	0.020	0.004	0.012	0.028	4.23%
Total effect	0.472	0.029	0.4156	0.5279	100%

SE = Standard error; Ind 1 = Life satisfaction → Family health → Health-promoting lifestyle; Ind 2 = Life satisfaction → Physical activity → Health-promoting lifestyle; Ind 3 = Life satisfaction → Family health → Physical exercise → Health-promoting lifestyle.

## 4 Discussion

This study used a chain mediation model to analyze the relationship between life satisfaction and health-promoting lifestyle among young adults in China. The results indicated that family health and physical activity play a partial mediating role in this process, further confirming the close link between life satisfaction and health-promoting lifestyles (21). Family health and physical activity are aspects that have often been overlooked in the past. These findings help us better understand the relationship between life satisfaction and health-promoting lifestyle and offer insights and directions for improving health-promoting lifestyle practices in future.

In this study, the Cronbach’s α coefficients for all scales ranged from 0.705 to 0.956, and there was no common method bias. This indicates that the results of this study can effectively identify the various characteristics of young adults in China. First, our findings confirm Hypothesis 1: life satisfaction among young adults in China is closely related to their health-promoting lifestyles, which is consistent with the findings of Choi (49). Despite significant achievements in economic development and social construction, China still faces severe economic inequality, educational inequality, and health inequality. In this context, these inequalities are considered major reasons why young adults strive for and compete for more material wealth (50). This also reduces the life satisfaction of young adults in China, leading to psychological and health problems (51). Life satisfaction reflects an individual’s overall sense of fulfillment and happiness in life, which is influenced not only by objective conditions but also by subjective perceptions (13).Cognitive Behavioral Theory (CBT) suggests that an individual’s cognition can positively predict their behavior (52, 53), including their health behaviors (8). In other words, when an individual’s life satisfaction changes, their health-promoting lifestyle also changes in a positive correlation. Therefore, life satisfaction among young adults in China may be a crucial factor in improving their health behaviors.

This study has verified the mediating role of family health between life satisfaction and health-promoting lifestyle, aligning with findings from Kim (11), that family health and life satisfaction can enhance individual’s health-promoting lifestyle. This result supports Hypothesis 2. Building upon this, our research further confirmed the positive predictive effect of life satisfaction on family health, suggesting that an increase in life satisfaction contributes to the improvement of overall family health and thereby promotes healthier behaviors among family members (54). In other words, a good level of family health can mediate the impact of life satisfaction on health-promoting lifestyles among young adults in China. The mechanism of this process can be explained by the Emotional Contagion Theory (ECM). The theory posits that within family members or the entire family, cognition and behavior are often transmitted through unconscious imitation, emotional synchronization, and empathy. Additionally, studies have shown that not only cognition but also behaviors can be effectively transmitted within families (55, 56). Considering the trend toward smaller family sizes, dual-income households, and shifting gender role perceptions in China (57, 58), the influence of family on individual behaviors is expected to grow, making family health an important area for further attention in our future work.

Our study also confirmed Hypothesis 3, which states that physical activity significantly mediates the relationship between life satisfaction and health-promoting lifestyles among young adults in China, consistent with the findings of other studies (59). Physical activity is one of the most effective and cost-efficient methods for enhancing health and is an important way to promote health (60). When an individual’s life satisfaction increases, it indicates a more stable psychological state, reducing the need to compete for material possessions. This allows them to balance work and rest, effectively relieving psychological stress (61). Under these conditions, individuals are more inclined to allocate their time reasonably, spending it on relaxation, social interactions, and physical activities (62). Wankel (63) demonstrated that individuals with higher positive emotions are more likely to engage in physical activities. These results suggest that when individuals have higher life satisfaction, physical activity acts as a crucial mediator that transforms cognitive health awareness into physical and mental health benefits. The underlying mechanism may be attributed to enhanced self-efficacy (64). In other words, the sense of achievement or positive psychological benefits gained from exercise, or the positive physiological responses experienced after exercise, such as increased strength and vitality, can motivate individuals to maintain this state through more comprehensive health behaviors.

Finally, our study found that life satisfaction can influence the health-promoting lifestyles of Chinese young adults by affecting their family health, which in turn impacts their physical activity. The construction of this model provides a new perspective for understanding how life satisfaction affects the health-promoting lifestyles of young adults. As previously mentioned, the positive affective value associated with high life satisfaction is a key factor motivating individuals to engage in health-promoting behaviors (7), this study further confirms the link between the two. Additionally, the study by Michaelson et al. (65) highlights the significant impact of family health on individual health behaviors and underscores the role of the family environment as a critical setting for health promotion. This link between cognitive and environmental changes and the transformation of individual behaviors also garners support from the Multi-Theory Model (MTM) (66). The MTM posits that life satisfaction and family health, as cognitive and environmental elements, are instrumental in fostering individual health behaviors, with physical activity acting as a mediating factor in this process (67, 68). In other words, an elevation in life satisfaction not only fortifies the sense of subjective well-being but also advances the overall health status of the family. Such an enhancement is likely to be reflected in increased care and support among family members, coupled with a heightened awareness of health. Consequently, this could foster a greater array of family-oriented activities, such as family sports events, which in turn could improve the health-promoting lifestyle of individuals. At the same time, within the cultural context of China, we believe that this family-based mediation effect is more stable.

## 5 Limitation

There are some limitations in this study, First, it is a cross-sectional study that only indicates correlations between variables without establishing causation. Secondly, all the questionnaires used in this study are self-reported. Since the questionnaire involves numerous questions about actual family situations, the results may be affected by participants’ self-defense. Finally, the sample size in this study is only from the southwestern region of China. Due to this regional limitation, the findings may not be representative of the young adult population nationwide. Therefore, future studies should consider employing longitudinal or experimental research designs to more precisely uncover the causal relationship between life satisfaction and health-promoting lifestyle. Simultaneously, the recruitment of study samples should be more representative to minimize potential sampling biases and strengthen the external validity of the research findings.

## 6 Conclusion

This study verified the mediating roles of family health and physical activity in the relationship between life satisfaction and health-promoting lifestyles among young people in China. It also confirmed the significant chain mediation effect among these variables. Our findings suggest that enhanced life satisfaction may be a potential avenue for improving health-promoting lifestyles among young adults in China, while the critical roles of family health and physical activity in this process warrant attention from scholars in the field. Moreover, it is expected that these results will garner the interest of policy makers and the academic community, prompting initiatives that aim to cultivate more healthful and cohesive family and social environments.

## Data Availability

The raw data supporting the conclusions of this article will be made available by the authors, without undue reservation.
